# More than What Meets the Eye: Differential Spatiotemporal Distribution of Cryptic Intertidal Bangiales

**DOI:** 10.3390/plants11050605

**Published:** 2022-02-24

**Authors:** Fernanda P. Cid Alda, Nelson Valdivia, Marie-Laure Guillemin

**Affiliations:** 1 Scientific and Technological Bioresource Nucleus (BIOREN), Universidad de La Frontera, Avenida Francisco Salazar 1145, Temuco 4780000, Chile; 2 Instituto de Ciencias Marinas y Limnológicas, Facultad de Ciencias, Campus Isla Teja, Universidad Austral de Chile, Valdivia 5090000, Chile; nelson.valdivia@uach.cl; 3 Centro FONDAP de Investigación de Ecosistemas Marinos de Altas Latitudes (IDEAL), Valdivia 5090000, Chile; 4 Instituto de Ciencias Ambientales y Evolutivas, Universidad Austral de Chile, Casilla 567, Valdivia 5090000, Chile; 5 CNRS, Sorbonne Université, IRL 3614, Evolutionary Biology and Ecology of Algae, Station Biologique de Roscoff, CS 90074, F-29688 Roscoff, France

**Keywords:** niche partitioning, co-occurring cryptic species, environmental gradients, Chile, *Fuscifolium*, *Porphyra*, *Pyropia*

## Abstract

Morphologically similar but genetically distinct species have been termed cryptic and most have been assumed to be ecologically similar. However, if these species co-occur at a certain spatial scale, some niche differences at finer scales should be expected to allow for coexistence. Here, we demonstrate the existence of a disjointed distribution of cryptic bladed Bangiales along spatial (intertidal elevations) and temporal (seasons) environmental gradients. Bladed Bangiales were identified and quantified across four intertidal elevations and four seasons for one year, at five rocky intertidal sites (between 39° S and 43° S) in southern Chile. Species determination was based on partial sequences of the mitochondrial cytochrome c oxidase 1 (COI) gene amplification. To assess species gross morphology, thallus shape, color, and maximum length and width were recorded. Hundreds of organisms were classified into nine Bangiales species belonging to three genera (i.e., *Fuscifolium*, *Porphyra*, and *Pyropia*), including five frequent (>97% of specimens) and four infrequent species. All species, except for *Pyropia saldanhae*, had been previously reported along the coasts of Chile. The thallus shape and color were very variable, and a large overlap of the maximum width and length supported the cryptic status of these species. Multivariate analyses showed that the main variable affecting species composition was intertidal elevation. Species such as *Py. orbicularis* were more abundant in low and mid intertidal zones, while others, such as *Po. mumfordii* and *Po.* sp. *FIH*, were principally observed in high and spray elevations. Despite all numerically dominant species being present all year long, a slight effect of seasonal variation on species composition was also detected. These results strongly support the existence of spatial niche partitioning in cryptic Bangiales along the Chilean rocky intertidal zone.

## 1. Introduction

The study of species co-occurring along environmental gradients allows us to improve our mechanistic understanding of biodiversity. For instance, cryptic species are commonly found co-occurring in organisms such as corals [1,2], nematodes [3], rotifers [4], and red algae [5,6] living in aquatic environments subjected to strong physical or chemical gradients (e.g., light intensity, desiccation stress, or salinity). Cryptic species have been classically defined as genetically distinct taxa that have been erroneously classified under a single nominal species name because they are, at least superficially, morphologically indistinguishable [7,8]. These cryptic taxa have been shown to be especially common in the marine realm [9]. Cryptic species that are morphologically similar have been assumed to be ecologically similar, which implies that they require very similar resources [7,8]. However, if these species are actually coexisting, some niche differences would be expected [1,2,9,10,11].

Contemporary coexistence theory predicts that niche partitioning allows for stable long-term coexistence of co-occurring species if their differences in competitive abilities (i.e., “fitness differences”) are not extremely large [12,13]. Therefore, cryptic species being truly ecologically similar and competing for the same resources should be observed in the same habitat only transiently [14,15]. Alternatively, Hubbell’s [16] unified neutral theory of biodiversity and biogeography posits that coexistence could be due to stochastic events of reproduction, death, and dispersal along with speciation but without any effect of niche partitioning or selection. Yet, such an extreme ecological equivalence between coexisting species has proven to be elusive in nature, and minor niche differences have been shown to underpin the slow competitive exclusion of inferior competitors (e.g., [17]). Cryptic species, despite largely similar morphologies, have been shown to exhibit subtle differential physiological tolerances, leading to niche partitioning and stable coexistence [4].

The rocky intertidal zone provides an ideal habitat to assess the association between the composition of cryptic species and environmental gradients over space and time. In the intertidal zone, stress increases with elevation, due to desiccation and sun exposure, favoring rapid vertical changes in community composition [18,19]. The strength of desiccation, jointly with other factors, such as the distribution of consumers, can affect the temporal and spatial distribution and composition of resource species (e.g., macroalgae [20,21]). Moreover, intertidal habitats in temperate biogeographic zones can be exposed to important seasonal variations in abiotic environmental conditions, affecting sessile invertebrate and macroalga performance and community structure [22,23,24,25,26]. These sharp environmental intertidal gradients offer a variety of environmental niches for locally co-occurring (i.e., a few meters) species. If intertidal cryptic species are not fully ecologically similar, therefore, we should expect a significant association between the occurrence and abundance (i.e., community composition) of cryptic species and environmental gradients over space and time [27].

Multiple macroalgae cryptic species have been identified co-occurring along rocky intertidal habitats. For example, cryptic species of the genus *Ectocarpus* (brown algae) show differences in attachment substrate and tidal zonation at a very small scale (i.e., a few meters [28,29]). Regarding other groups of cryptic macroalgae, such as bladed Bangiales (red algae), only a few studies have been performed along the intertidal gradient. In New Zealand, nine species were recorded along a three-year study in a single location, but no differences in seasonal growth or small-scale distribution were detected between the two most common species [30]. In central Chile, a small-scale study conducted at Maitencillo beach reported the presence of the two most common cryptic Bangiales (i.e., *Porphyra luchea* and *Pyropia variabilis*) across several types of habitats, such as mid and high intertidal habitats and rocky platforms [31]. However, *Porphyra longissima* was restricted to rock boulders surrounded by sand in Maitencillo, a habitat where no other species were recorded, hinting at a possible differentiation of ecological niches among bladed Bangiales [31]. This idea is supported by the slightly distinct gene expression and ecophysiological responses of *Pyropia orbicularis* and *Py. variabilis* to desiccation [32]. Similarly, variations in ecophysiological responses have been used to explain differences in species distribution among cryptic red algae of the genus *Bostrychia* [33]. In addition, strong differences in the temporal patterns of appearance and growth were detected between the seven species of bladed Bangiales present in New Hampshire (USA), with some aseasonal species present all year round (as *Po. ‘umbilicalis’*), while others were highly seasonal (as *Po. ‘yezoensis’*) [34]. Taken jointly, these previous studies suggest that ecophysiological differences and/or differences in competitive abilities could explain the long-term coexistence of groups of closely related cryptic bladed Bangiales along temperate coasts.

In Chile, as in other part of the word, bladed Bangiales have attracted considerable interest because of their ecological and economical importance. Indeed, this group includes species commonly known as “nori”, which represent the most valuable macroalgal crop, with a market value estimated to be more than USD 1.3 billion per year in China, Japan, and Korea [35]. In Chile, the bladed Bangiales are commonly known as “Luche”. They are harvested and sold fresh or dry for local consumption and represent an important economic resource for the Chilean artisanal fishermen [31,36,37]. A first genetic analysis carried out along the southeastern Pacific coast has uncovered a high number of genetically distinct taxa, most of them still unnamed [6]. Chilean Bangiales lack conspicuous diagnostic morphological differences that could allow unambiguous species determination. Indeed, even when taking into account microscopic and macroscopic traits and the developmental features of reproductive tissues, morphology did not allow the four more common species sampled in Maitencillo to be distinguished effectively [31]. Accurate species identification based on morphology is extremely difficult, which explains why Chilean Bangiales have been misidentified for decades. For example, many specimens sampled along the Chilean coast were, for many years, incorrectly assumed to be *Porphyra columbina*, a species that we now know to be endemic of the sub-Antarctic waters of New Zealand [38,39]. Moreover, critical basic information, such as the ecological requirement for growth and reproduction, is still lacking for these species, limiting the development of aquaculture programs for these important resources for both human food and new bioproducts.

Here, we test the hypothesis that niche differences will lead to distinct spatiotemporal patterns of several cryptic bladed Bangiales along spatial (intertidal elevations) and temporal (seasons) environmental gradients. As an alternative hypothesis, we predict that ecological similarity and, thus, competitive exclusion due to fitness differences generates transient patterns in the abundance of these species. Under a neutral scenario, with species lacking major niche and fitness differences, a random distribution of cryptic bladed Bangiales along intertidal elevations and seasons of the year is expected. To test these hypotheses, bladed Bangiales were collected from five rocky intertidal sites (platforms and/or boulders located between the 39° S and 43° S) across four intertidal elevations and four seasons for one year. Cryptic species determination was based on partial sequences of the mitochondrial cytochrome c oxidase 1 (COI) gene and we assembled a large dataset of morphological attributes and molecular data for these key primary producers of the Chilean intertidal zone.

## 2. Results

**Figure 1:**
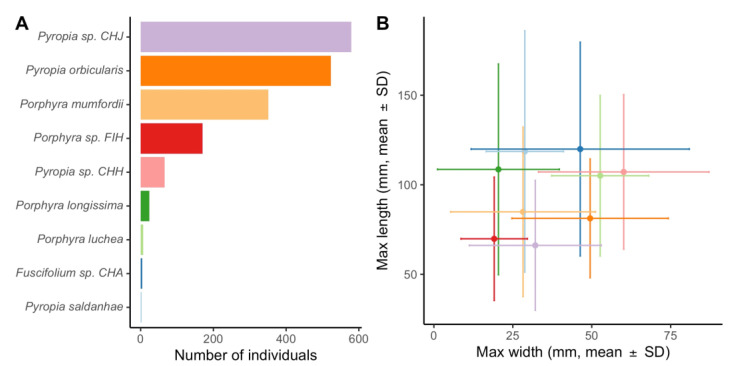
(**A**) Total number of individuals identified through COI sequencing to one of the nine Bangiales species detected in our study area. (**B**) Max length (mm ± SD) and max width (mm ± SD) for each of the nine Bangiales species studied. Number of individuals measured per species: *Pyropia* sp. CHJ (*n* = 356), *Pyropia orbicularis* (*n* = 224), *Porphyra mumfordii* (*n* = 204), *Porphyra* sp. FIH (*n* = 63), *Pyropia* sp. CHH (*n* = 60), *Porphyra longissima* (*n* = 21), *Porphyra luchea* (*n* = 7), *Fuscifolium* sp. CHA (*n* = 4), and *Pyropia saldanhae* (*n* = 3). Only complete wet thalli were measured. See the text for more details.

A total of 1990 individuals were sampled. No Bangiales could be observed in the spray-elevation zone during summer in Niebla (two unsampled spray-elevation plots; five samples each). Of these 1990 individuals, 1727 (i.e., 87%) were identified at the species level using COI sequences. For the remaining 263 individuals, low-quantity and/or low-quality DNA extraction did not allow for COI amplification and sequencing. Nine Bangiales species were identified (Figure 1A). The most abundant species were *Pyropia* sp. CHJ (579 individuals, 34% of the sequenced Bangiales), *Pyropia orbicularis* (523 individuals, 30% of the sequenced Bangiales), *Porphyra mumfordii* (351 individuals, 20% of the sequenced Bangiales)*, Porphyra* sp. FIH (170 individuals, 10% of the sequenced Bangiales), and *Pyropia* sp. CHH (66 individuals, 4% of the sequenced Bangiales). Four rare species of Bangiales were detected in our study area: *Porphyra longissima* (24 individuals identified), *Porphyra luchea* (7 individuals identified), *Fuscifolium* sp. CHA (4 individuals identified), and *Pyropia saldanhae* (3 individuals identified) (Table 1).

These 4 species corresponded to less than 2.5% of the sequenced Bangiales. No clear difference in gross morphology was detected between the nine Bangiales species for which measures of maximum width and length largely overlapped (Figure 1B). In the most abundant species, *Po. mumfordii*, *Po.* sp. FIH, and *Py. orbicularis*, thallus shape was very variable, with specimens showing lancelolate, elongated rosette, and rosette-like blades. Some species, such as *Py.* sp. CHH and *Py.* sp. CHJ, were characterized by more rosette-like or elongated rosette blades. The highest homogeneity in terms of thallus shape was observed in *Po. longissima*, for which 19 of the 21 measured specimens were characterized by lancelolate blades. Most sampled specimens were of brownish color. However, thallus color was highly variable in *Po. longissima* and *Py.* sp. CHJ, where thalli varied from red to brown, green, and yellowish, depending on the sampled specimen (Supplementary Table S1).

**Figure 2:**
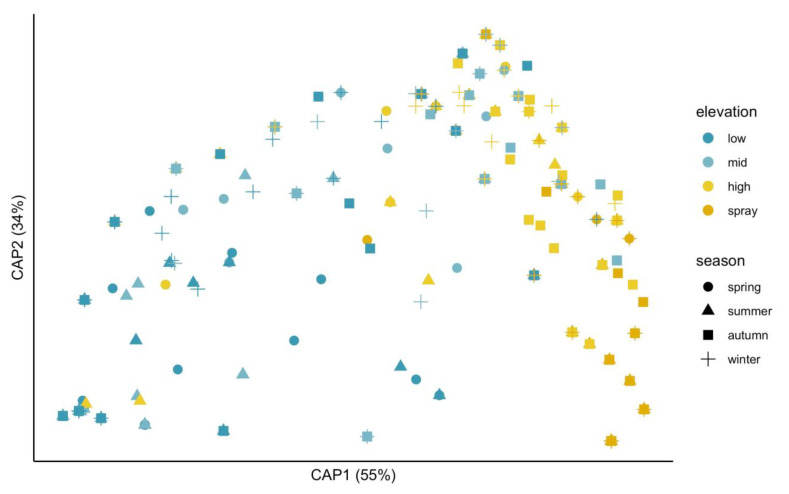
Principal coordinates analysis (PCoA) showing changes in species composition depending on the elevation and season. The first two axes explain more than 89% of the total variance.

The first axis of the principal coordinates analysis (PCoA) separated the samples according to intertidal elevation, with low- and mid-elevation samples located on the left and high- and spray-elevation samples located on the right of the axis (Figure 2). Nevertheless, a relatively high overlap between both groups was observed, in particular for the mid-elevation samples obtained during autumn (light-blue squares in Figure 2). Accordingly, the global PERMANOVA showed an elevation by season interactive effect on species composition (Table 2, *R*^2^ = 0.31). Pairwise comparisons indicated that species composition significantly varied along the vertical intertidal stress gradient across the year (Table 2). The exception was summer, when species composition at the mid and low elevations did not vary statistically (Table 2, *p* = 0.339).

**Figure 3:**
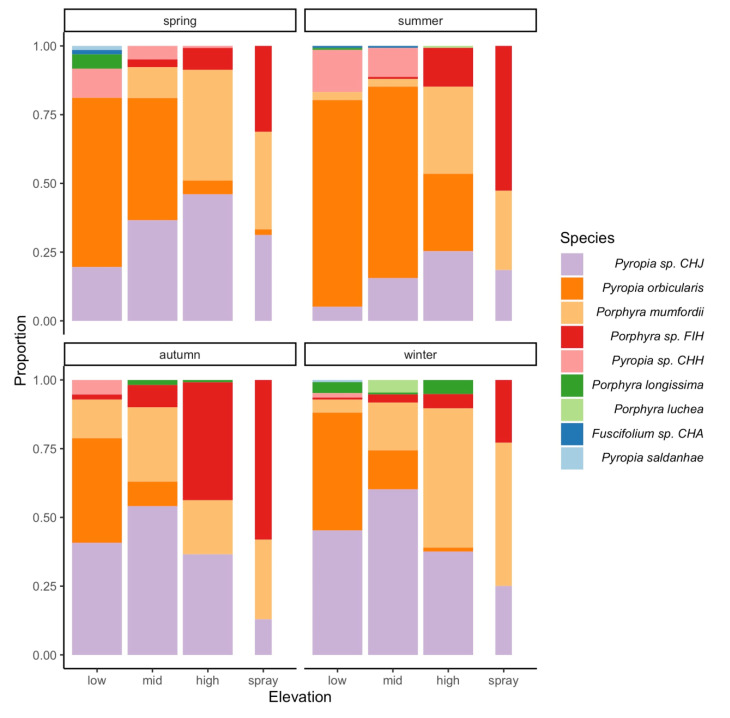
Proportion of Bangiales species identified at each intertidal elevation level (low, mid, high, and spray) during the four seasons. Bar width is proportional to number of samples at each intertidal elevation.

The abundance of *Py.* sp. CHJ increased from low to high intertidal elevations in spring and summer but decreased along the same environmental gradient during autumn and winter (Figure 3; see also post-hoc comparisons in Table 3). Elevation and season accounted for 27% of the spatial variation in the abundance of *Py.* sp. CHJ (*R*^2^*_c_* = 0.27). The entire model, including sampling sites, accounted for 40% of the variation in the abundance of this species (*R*^2^*_m_* = 0.40). The model significantly fit the observed data of *Py.* sp. CHJ abundance (LRT: *χ*^2^_16_ = 179.67, *p* < 0.001). *Pyropia orbicularis* decreased in abundance from low to spray elevations (Figure 3). The magnitude of this variation, however, differed among seasons, with broader variations observed during spring and summer than autumn and winter (Figure 3; Table 3). The fixed factors accounted for 89% of the variation in *Py. orbicularis* abundance and the entire model explained an 89.5% of the variation, indicating a small effect of the differences among sites on the abundance of this species (*R*^2^*_c_* and *R*^2^*_m_* = 0.890 and 0.895, respectively; LRT: *χ*^2^_16_ = 402.9, *p* < 0.001). The abundance of *Po. mumfordii* tended to increase with increasing intertidal vertical environmental stress across the year (Figure 3). These differences, however, were statistically significant only between the high and low elevations during summer and winter (Table 3). Accordingly, *R*^2^*_c_* and *R*^2^*_m_* were 0.88 and 0.96, respectively (LRT: *χ*^2^_16_ = 167, *p* < 0.001). *Porphyra* sp. FIH increased in abundance from low to spray elevations (Figure 3). The species was absent at low elevations during spring and summer and occurred with minimal abundances at low elevations during autumn and winter (Figure 3). The statistical model significantly fit the observed data of *Porphyra* sp. FIH abundance (*R*^2^*_c_* and *R*^2^*_m_* = 0.96 and 0.99, respectively; LRT: *χ*^2^_16_ = 108.4, *p* < 0.001). The comparatively low abundances of *Porphyra* sp. FIH resulted in statistically significant comparisons only during autumn (Table 3). *Pyropia* sp. CHH occurred mainly at low and mid elevations in spring and summer (Figure 3; *R*^2^*_c_* and *R*^2^*_m_* = 0.57 and 0.78, respectively; LRT: *χ*^2^_16_ = 145.73, *p* < 0.001). As above, the low abundances of this species led to a statistically non-significant post-hoc test (Table 3).

## 3. Discussion

Using the molecular and morphological data of hundreds of specimens that were collected seasonally in the southern part of the Chilean coast (39–43° S) for one year, we were able to detect patterns of niche partitioning among cryptic bladed Bangiales along the intertidal gradient. Indeed, differences in species composition according to intertidal elevation were detected, with *Pyropia orbicularis* being more abundant in the low and mid intertidal zones and *Porphyra mumfordii* and *Porphyra* sp. FIH being more abundant in the high and spray elevations. In total, we identified nine Bangiales species belonging to three genera (i.e., *Fuscifolium*, *Porphyra,* and *Pyropia*) in the study area, including five dominant (i.e., those species accounted for >97% of the sequenced specimens) and four infrequent species. Measurements of maximum thallus width and length showed overlapping patterns of gross morphology, supporting the cryptic status of these nine Bangiales species. Excepting *Porphyra longissima,* which was characterized by more lanceolate thalli, thallus shape was highly variable between the specimens of the same species, confirming a high intraspecific morphological variability. Even if we detected variation in community composition between seasons, all dominant species were a-seasonal and present all year long in our study area. As hypothesized for other cryptic species, we propose that niche partitioning could allow for the stable long-term coexistence of morphologically similar co-occurring species of bladed Bangiales.

### 3.1. Responses of Bladed Bangiales to Vertical Stress Gradients in Intertidal Habitats

Our results support the hypothesis that niche differences lead to distinct spatiotemporal patterns of cryptic bladed Bangiales in the southern part of the Chilean coast. Intertidal elevation accounted for most of the variation in species composition. Thus, and despite the observed overlaps in species abundances, our results support the existence of spatial niche partitioning in cryptic Bangiales along the intertidal zone.

The constant alternation of low and high tides determines that high intertidal elevations are characterized by strong physiological constraints for organisms living in intertidal habitats in terms of desiccation [40], high temperatures [41], nutrient shortages [42], and high UV radiation [43,44]. Thus, the upper distribution boundary of intertidal species is related to species physiological limits. Contrastingly, the lower limit of intertidal species distribution seems to be mainly regulated by biological interactions (e.g., consumption and competition) [45,46,47,48]. In southern Chile, for instance, small-sized grazers have been shown to strongly control the growth of intertidal macroalgae and sessile invertebrates over succession [49,50]. Thus, the interplay of biotic and abiotic factors generates a sharp gradient of environmental conditions and a broad niche space in intertidal habitats [51,52,53].

Even though bladed Bangiales were morphologically very similar, some species occurred at different intertidal elevations. For example, while *Py. orbicularis* was mainly present at low/mid-intertidal elevations, both *Po. mumfordii* and *Po.* sp. FIH were distributed higher on the shore. These patterns were not detected in the previous studies focusing on the center part of the Chilean coast [31,32,39,54]. The species *Py. orbicularis* was first described as colonizing the upper and mid intertidal area of the Maitencillo beach (central Chile, [39]), but the species’ abundance across intertidal elevations was not estimated in that study. In the same site, various studies also reported *Py. orbicularis* as commonly encountered in the high intertidal elevation [31,32]. It is possible that ecotypes with distinct preferences in intertidal elevations exist within *Py. orbicularis*, a species for which clear genetic differences have been detected between the samples from extreme south, south, central and north Chile [6]. In the same way, *Po. mumfordii* was reported from the low to the upper intertidal elevations in Montemar (central Chile, [54]) but without any estimation of the species abundance. However, and confirming our results, higher abundances of *Po. mumfordii* at high intertidal elevations have also been observed in the northern hemisphere [55,56].

As for most bladed Bangiales, Chilean species have been classically described as extremely resistant to the stressing conditions characteristic of the high intertidal zone (e.g., desiccation, temperature, and UV; [40,44,57,58,59]) and the existence of species-specific tolerance limits acting as key determinants of the upper vertical limits of the distribution in these species is still unclear. For example, experimental studies have shown that Chilean bladed Bangiales are highly tolerant to desiccation, being able to lose more than 90% of their water contents and recover cellular activities after only five minutes of rehydration [59]. Moreover, these species present a low sensitivity to UV radiation and a rapid recovery from solar stress [44]. In other coastal regions, differences in physiological response to stress have been detected between Bangiales species occupying distinct tidal levels. Indeed, strong differences have been detected between the high- and low-intertidal species in terms of nutrient uptake and growth rate (northwest Atlantic; [60,61]), membrane damage (North East Pacific; [41]), and photosynthetic activities (North West Pacific, [62]). Collectively, these works suggest that fine-scale niche partitioning between bladed Bangiales species, mostly due to dissimilarities in physiological tolerance to stress, can generate distinct distribution patterns. These results have also been confirmed in filamentous Bangiales species for which distinct adaptation to temperature and salinity could explain, at least in part, species distribution [63]. Whether the cryptic bladed Bangiales identified in our study respond differently to abiotic environmental factors is still an open question, and further manipulative experiments are now needed to understand the physiological responses to environmental stress in these species assemblages.

### 3.2. Seasonal Variation in Intertidal Species Assemblages

Even if the effect of seasonal variation on species composition was much weaker than that imposed by intertidal elevation, our results show that seasonal changes affect bladed Bangiales communities in south Chile. For example, the distribution patterns observed for *Py.* sp. CHJ varied between seasons, with an increase in abundance from low to high intertidal elevations in spring and summer but a decrease along the same environmental gradient during autumn and winter.

Nonetheless, all the relatively abundant species observed in the study area were present across the four seasons sampled. While we might need a multiyear sampling of species occurrences and abundances to confirm these results, the present study suggests that the assemblage of species is very persistent over time (i.e., there are no transient species). The relatively strong temporal persistence of the species assemblage observed in our study contrasts with previous studies demonstrating the existence of both persistent and transient cryptic bladed Bangiales in intertidal habitats (e.g., New Hampshire, USA [34]; New Zealand [30]. For rare species, it is much harder to conclude on a possible persistent or transient status. However, even for species for which only a few specimens were sampled, no clear seasonal patterns were detected. For example, *Po. longissima* (24 specimens collected in total) was observed during the four seasons, *Po. luchea* (6 specimens collected in total) was observed during both winter and summer, *F.* sp. CHA (4 specimens collected in total) was observed during spring and summer, and *Py. saldanhae* (3 specimens collected in total) was observed during spring and winter. In northern Atlantic populations of *Po.* *umbilicalis*, the production of spores (i.e., asexual spores or neutral spores) has been shown to be highly seasonal, with a higher spore release from fall to early spring [57,64,65]. These results are concordant with observations of the bladed Bangiales recruitment peak during autumn in New Zealand [30]. It is possible that most species of Chilean bladed Bangiales also reproduce during the colder periods, affecting the cover and biomass of Bangiales within each site but not necessarily the structure of the entire species assemblage [65].

### 3.3. Diversity and Biogeography of Bladed Bangiales

In the present study, measures of maximum blade width and length exhibited an overlap between the nine identified species. These results support the cryptic status of these species, at least at the level of gross morphology. Similar results were obtained for the bladed Bangiales species community located in central Chile, where only *Po. longissima* could be clearly differentiated morphologically from the rest of the bladed Bangiales, due to its long and thin blades [31]. At our study sites, *Po. longissima* was also characterized by long, thin, lanceolate thalli. However, *Po. longissima* shared these characteristics with some specimens from other species. A high level of variability in terms of thallus color, shape, and size was observed within species, probably due to phenotypic plasticity. A previous study conducted in central Chile detected clear phenotypic plasticity in *Po. mumfordii*, with specimens from the high and mid intertidal habitats showing long, thin, lanceolate thalli, while the ones from the low intertidal habitats were characterized by much wider elongated rosettes [54]. Taken together, these results are in agreement with the previous studies that showed that morphological characters alone could lead to inaccurate species determination in bladed Bangiales [66,67,68,69]. In many taxa, most detected cryptic species represent recently diverged entities that still share the same gross morphology [70]. However, molecular studies have also revealed the existence of non-monophyletic complexes of cryptic species, and morphological similarities in these cases have been associated with evolutionary convergence, morphological stasis, or developmental constraints [70]. In our species assemblage, *Py.* sp. CHJ and *Py. orbicularis* have been shown to be close genetic groups [6] and could represent recently diverged species that have not accumulated any morphological differences yet. However, the nine species sampled in the present study are part of three very divergent genera (i.e., *Fuscifolium*, *Porphyra*, and *Pyropia*; [68]), and the evolutionary convergence or morphological stasis linked to life in the highly stressful intertidal zone (e.g., [71]) could be hypothesized in bladed Bangiales.

Eight of the nine species sampled in our study sites had previously been reported in Chile, with six of them sampled along the southern part of the coast [6]. Two rare species, *F.* sp. CHA and *Po. luchea*, were previously known only in the northern part of Chile (*F.* sp. CHA was reported in Puerto Oscuro, 31° S and *Po. luchea* was reported in Maitencillo, 32° S and Chañaral de Aceituno, 29° S; [6,31]). Thus, our results demonstrate that the distribution areas of both *F.* sp. CHA and *Po. luchea* extend well into the southern part of the country. Another dominant and commonly encountered species, *Po. mumfordii*, also presents a much wider distribution than the one proposed by Guillemin and collaborators (i.e., reported from 39° S up to 41° S, 2016), extending between 8° S Salaverry, Peru [72], and 43° S (Melinka, Chile, present study).

The present study is the first report of *Py. saldanhae* in South America, a species considered to be endemic to the west coast of South Africa and Namibia [73,74,75]. Genetic data show that *Py. saldanhae* forms a well-supported clade with various species of the Falkland Islands and New Zealand, and the authors suggested that *Py. saldanhae* has speciated along the South African coasts after a Pleistocene-Pliocene west-to-east colonization event [75,76]. Actually, rafting-mediated transport along the Antarctic Circumpolar Current has been shown to favor macroalgae dispersal between isolated landmasses in the Southern Ocean [77,78,79]. The presence of *Py. saldanhae* in south Chile, though, opens questions about the possible origin of this population. Even if we cannot completely rule out a recent introduction by human marine transport, the geographical position of our study region does not link to any major South Africa to south east Pacific shipping routes and is not located near any major Chilean harbor. It is also possible that the species is locally rare but presents an ample distribution, including the Chile Patagonia, Falkland Islands, and South Africa. Renewed efforts, including extensive sampling in the cold waters of the southern hemisphere and the acquisition of a multigenic data set, are now needed to better resolve the evolutionary history of the bladed Bangiales in the region.

## 4. Materials and Methods

### 4.1. Study Area

**Figure 4:**
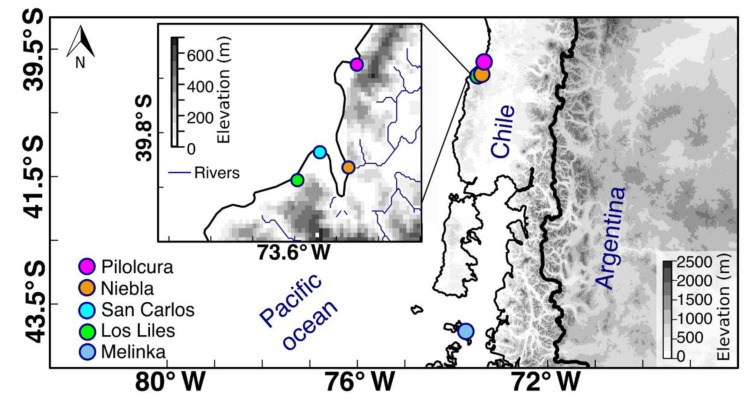
Location of the five sampling sites with Pilolcura (pink), Niebla (orange), San Carlos (light blue), and Los Liles (green) situated at the mouth of the Valdivia River estuary and Melinka (dark blue) in Ascension Island.

Samples were collected during each season in five sampling sites: San Carlos (39°51′46.52″ S/73°26′28.24″ W), Los Liles (39°53′31.19″ S/73°29′6.42″ W), Niebla (39°52′6.97″ S/73°24′7.05″ W), Pilolcura (39°40′20.54″ S/73°21′10.72″ W), and Melinka (43°53′ 56.13″ S/73°44′13.41″ W). The first four sites are located in the Los Ríos region while Melinka is located in the Aysén region (Ascension Island; Figure 4). Both regions are separated by approximately 500 km (Figure 4).

Abiotic environmental data were downloaded from Bio-ORACLE [80,81] (www.bio-oracle.org, accessed on 4 January 2022; Supplementary Table S1 in the Supplementary Materials). Present-day climate layers were obtained at a 5 armin spatial resolution. The maximum surface current velocity ranged from 0.398 m s^−1^ (San Carlos and Los Liles) to 0.095 m s^−1^ (Melinka, Supplementary Table S1). The maximal salinity was relatively higher in Los Ríos (between 34.12 and 34.14 PSU) than in Aysén (33. 03 PSU). The maximal surface seawater temperature was highest at Pilolcura (15.23 °C, Los Ríos) and lowest at Melinka (13.88 °C, Aysén). The maximal photosynthetically active radiation (PAR) peaked at Niebla (64.02 μE m^−2^ d^−1^) and was lowest at Melinka (47.59 μE m^−2^ d^−1^; Supplementary Table S1).

The sites in the Los Ríos region correspond to wave semi-exposed rocky shores. However, water circulation has been reported as highly complex in the area, due to the effect of local upwelling, freshwater plumes, and offshore eddies [82]. Two of the four sites, San Carlos and Niebla, are located at the mouth of the Valdivia River. Los Liles and Pilolcura, on the other hand, correspond to more open costal habitats [83].

In the Aysén region, Ascension Island is part of the large archipelago of Las Guaitecas, an area characterized by an intricate coastline with many small islands separated by channels. Melinka is located along the island’s coast facing the Corcovado gulf and represents a fairly protected area from the prevailing winds, flowing from west to east (see also Supplementary Table S1).

Rocky intertidal habitats in both study regions are dominated in terms of biomass by sessile species, such as the acorn barnacles *Jehlius cirratus* and *Notochthamalus scabrosus* (high intertidal), the purple mussel *Perumytilus purpuratus*, the red corticated alga *Mazzaella laminarioides* (mid intertidal), and large brown kelps, such as *Lessonia spicata*, *Durvillaea incurvata*, and *Macrocystis pyrifera* (low intertidal) [44,84,85,86]. The assemblage of consumers comprises macrograzers, such as *Fissurella picta, Chiton granosus,* and *Scurria zebrina*, and mesograzers, such as the pulmonate gastropod *Siphonaria lessoni*, littorinids, and amphipods [84,86]. Mesograzers are particularly abundant in the Los Ríos region, likely stemming from the overexploitation and functional extinction of predators and larger grazers; consequently, mesograzers have been demonstrated to exert a strong top-down control on the assembly of sessile communities [50,87].

The samplings were conducted between late May and early June 2020 for the autumn season, between late July and early August 2020 for the winter season, during December 2020 for the spring season, and between late February and early March 2021 for the summer season. A slight difference in sampling timing was due to the limited access to Ascension Island, especially during bad weather conditions.

### 4.2. Bladed Bangiales Sample Collection

**Figure 5:**
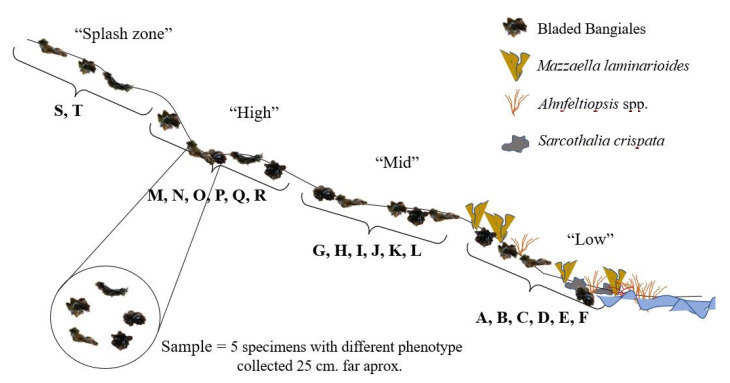
Intertidal zonation of bladed Bangiales sampling. The four levels that were sampled (“splash zone”, “high”, “mid”, and “low”) are indicated. Capital letters correspond to samples encompassing 5 nearby specimens collected in a 25 cm radius. See the text for more details.

Samples were taken from rocky platforms and boulders within four intertidal elevations (Figure 5). The upper and lower limits of the *Mazzaella laminarioides* belt were used as the major local biological markers to define the distinct intertidal levels sampled (Figure 5). The first intertidal level, named “low”, corresponded to samples encountered mixed with species such as *Ahnfeltiopsis* spp. or *Sarcothalia crispata* and completely embedded within the *M. laminarioides* belt. These samples were located at the limit between the midlittoral and infralittoral zones and generally corresponded to the bladed Bangiales specimens observed lowest at the shore. The second level, named “mid”, corresponded to samples encountered at the upper limit of the *M. laminarioides* belt or slightly higher. The third level, named “high”, corresponded to samples located at the limit between the supralittoral and midlittoral zones, where no *M. laminarioides* were observed. The fourth level, named “splash zone”, corresponded to the highest elevation of the supralittoral zone where bladed Bangiales could still be found. Samples were collected by group of five nearby specimens, all located within a circle of 25 cm and bagged together. When possible, neighboring specimens presenting different phenotypes (i.e., distinct color, form, or size) were collected. For the first three levels, six samples were collected and noted A to F, corresponding to “low”, G to L to “mid” and M to R to the “high” intertidal levels (Figure 5). For the “splash zone” two samples were collected, noted S and T. The samples collected within the same level were separated by at least 2 m. When available, at each season and in each locality, 100 algae specimens were collected in total (i.e., 20 samples of five specimens each).

Algal blades were spread flat to the best of our ability and photographed jointly with a size scale before cutting a small portion of tissue that was then stored in silica gel for further molecular work. Only complete blades, still wet, were photographed.

### 4.3. Morphological Traits

Using the photographs taken of the blades spread flat, the maximum lengths and maximum widths of the thalli were measured using the software ImageJ (version Java 1.8.0) (http://imagej.nih.gov/ij/, accessed on 24 May 2021). Some other characteristics such as color (brown, green, yellow, and red), and form (rosette, elongated rosette, and lanceolate blade) were also noted.

### 4.4. Species Determination Based on Molecular Data

Algal material was subjected to cell disruption for 30 s in a Mini-BeadBeater (BioSpec Inc., Bartlesville, OK, USA) and DNA was extracted using the Plant DNA kit (Omega BIO-TEK, Norcross, GA, USA) following the provider’s protocol. Quantity and quality of the extracted DNA was determined using a NanoDrop Lite spectrophotometer (Thermo Scientific, Waltham, MA, USA). A fragment of the mitochondrial cytochrome c oxidase 1 (COI) gene was amplified using the primer pair GazF1 (5′-TCA ACA AAT CAT AAA GAT ATT GG -3′) and GazR1 (5′-ACT TCT GGA TGT CCA AAA AAY CA -3′) following the PCR protocol described by Saunders [88], with a melting temperature of 56 °C. The PCR products were checked using 1.5% agarose electrophoresis gels. Sanger sequencing was performed using the GazF1 primer in the AUSTRAL-omics Core-Facility (Universidad Austral de Chile). The sequences were checked by hand using Chromas v.2.6.6 (http://technelysium.com.au/wp/chromas/, accessed on 10 May 2021), and species identification was performed using the GenBank Basic Local Alignment Search Tool (BLAST) to compare the newly obtained COI sequences with the ones already deposited in the public databases in NCBI GenBank (https://www.ncbi.nlm.nih.gov/, accessed on 20 December 2021). Species determination was based on the percent identity BLAST metric and a minimum identity threshold of 3% was used (i.e., reflecting the congeneric interspecific divergences observed for Chilean bladed Bangiales for the COI) [6]. The nucleotide sequences obtained in this study were deposited in the GenBank database; all accession numbers are available in the Supplementary Table S2.

### 4.5. Statistical Analyses

Composition of cryptic species (i.e., the combination of species occurrences and abundances) was analyzed with permutational multivariate analyses of variance (PERMANOVA; [89]. The model included intertidal elevation (low, mid, high, and spray level) and season (spring, summer, autumn, and winter) as fixed and crossed factors. The species abundance data (number of individuals) were transformed to Bray–Curtis dissimilarities before the analysis, and 999 permutations of raw data were constrained within sites (Pilolcura, San Carlos, Niebla, Los Liles, and Melinka). After fitting the global model, we conducted between-elevation PERMANOVA post-hoc pairwise comparisons within each season. To control the type I error rate, we used a “treatment” contrast in which each elevation was compared against the low-tide elevation (reference group). Moreover, the spatial patterns of the community structure were described in a constrained principal coordinates analysis (PCoA) ordination [90]. This method allowed us to show the multivariate axes that accounted for most of the variation in the species abundance dataset according to our predictive model (i.e., separate and joint elevation by season effects on species composition).

Individual counts of numerically dominant species were separately analyzed with zero-inflated hierarchical generalized linear models. The error structure was modeled as a negative binomial distribution and model parameters were estimated by means of maximum likelihood. The models included elevation and season as fixed and crossed factors and site as a hierarchical factor. Model goodness-of-fit was assessed by means of conditional and marginal pseudo-coefficients of determination (*R*^2^*_c_* and *R*^2^*_m_*, respectively; [83,84]). Conditional *R*^2^*_c_* represents the fit of the fixed factors (elevation and season); marginal *R*^2^*_m_* represents that of the entire model (including the random effects). Plots of fitted vs. observed values were used as model diagnostics. Statistical fit was tested with a likelihood ratio test (LRT) in which a chi-squared test (*χ*^2^) was used to contrast the explained deviance of the model against that of a null model that included only the intercept. After fitting the global models, we conducted Tukey-corrected post-hoc comparisons. As above, we used a “treatment” contrast with low elevation as a reference group.

The vegan R package was used for running PERMANOVA and PCoA, glmmTMB was used for the zero-inflated models, MuMIn was used for pseudo-R^2^, and the tidyverse, cowplot, RColorBrewer, and wesanderson packages were used for data preparation and plotting [90,91,92,93,94,95,96,97,98,99,100]. All statistical analyses were conducted in R version 3.0.3 [93].

## 5. Conclusions

Our study provides useful insights into the mechanistic understanding of the growing diversity of cryptic species. The observed differences in the occurrence and abundance of cryptic bladed Bangiales along the rocky intertidal zone in south Chile hint at niche partitioning within this assemblage. This, in turn, has probably favored the co-existence in time of these Bangiales species and indicates that they are not fully ecologically similar. Future research on physiological responses to biotic and abiotic environmental stress would help us to understand the mechanisms underpinning the observed spatial patterns. In bladed Bangiales, as in other species characterized by a complex life cycle, both the microscopic sporophyte (also known as the conchocelis phase) and the macroscopic gametophytic phases are important to study. Indeed, laboratory-based experimental studies have shown that the sporophyte and gametophyte phases respond differently to abiotic stresses (light and temperature [101,102]) and grazing pressure by intertidal mollusks [103]). We suggest that similar morphologies do not equal similar environmental tolerances, which challenges the widely accepted use of comparative anatomy to define functional types, e.g., [104] and, thus, to understand how individual species influence the community structure and ecosystem functioning.

## Figures and Tables

**Table 1:** Raw counts of the species identified in the five sites sampled for each season.

		*Pyropia* sp. *CHJ*	*Pyropia orbicularis*	*Porphyra mumfordii*	*Porphyra* sp. *FIH*	*Pyropia* sp. *CHH*	*Porphyra longissima*	*Porphyra luchea*	*Fuscifolium* sp. *CHA*	*Pyropia saldanhae*	Ʃ
Autumn	Pilolcura	28	23	2	27	4	0	0	0	0	84
	Niebla	48	11	8	0	2	3	0	0	0	72
	San Carlos	15	3	42	18	0	0	0	0	0	78
	Los Liles	44	3	23	19	0	0	0	0	0	89
	Melinka	16	13	2	13	0	0	0	0	0	44
Winter	Pilolcura	42	20	0	7	1	3	0	0	0	73
	Niebla	39	18	31	0	1	3	0	0	0	92
	San Carlos	53	5	15	4	0	7	0	0	1	85
	Los Liles	55	2	30	8	0	0	0	0	0	95
	Melinka	10	30	45	3	0	0	6	0	0	94
Spring	Pilolcura	29	48	0	11	1	0	0	0	0	89
	Niebla	55	11	0	0	21	5	0	0	0	92
	San Carlos	33	28	27	0	0	2	0	1	2	93
	Los Liles	40	8	25	17	0	0	0	1	0	91
	Melinka	0	58	37	2	0	0	0	0	0	97
Summer	Pilolcura	12	76	0	1	1	0	1	1	0	92
	Niebla	23	25	1	0	35	0	0	0	0	84
	San Carlos	4	64	7	20	0	0	0	0	0	95
	Los Liles	31	21	14	20	0	1	0	1	0	88
	Melinka	2	56	42	0	0	0	0	0	0	100
Ʃ		579	523	351	170	66	24	7	4	3	1727

**Table 2:** Results of the PERMANOVA on the effects of intertidal elevation and season on Bangiales species composition. Pairwise tests were performed within each season with the low-tide elevation used as the reference group for the comparisons between the intertidal elevations.

Effect	df	F	R^2^
Elevation	3	42.91 **	0.19
Season	3	15.82 **	0.07
Elevation: season	9	3.59 **	0.05
Residuals	464		0.68
Total	479		1
Pairwise test			
Season	Intertidal elevation		
Spring	Mid **		
Spring	High **		
Spring	Spray **		
Summer	Mid		
Summer	High **		
Summer	Spray **		
Autumn	Mid **		
Autumn	High **		
Autumn	Spray **		
Winter	Mid **		
Winter	High **		
Winter	Spray **		

df = degrees of freedom; F = F ratio; R^2^ = coefficient of determination; Asterisks denote statistical significance: ** *p* < 0.01.

**Table 3:** Tukey-corrected post-hoc comparisons after the zero-inflated hierarchical generalized linear models. All comparisons were conducted with a “treatment” contrast with low elevation as the reference group and 462 degrees of freedom. The t-ratio of each comparison is shown.

Season	Intertidal Elevation	*Pyropia* sp. CHJ	*Pyropia orbicularis*	*Porphyra mumfordii*	*Porphyra* sp. FIH	*Pyropia* sp. CHH
Spring	Mid	2.40	−1.5	<−0.01	<−0.01	−1.5
Spring	High	3.16 **	−6.2 ***	<−0.01	<−0.01	−2.5
Spring	Spray	−1.31	−4.3 ***	<−0.01	<−0.01	<−0.01
Summer	Mid	2.3	−0.31	<−0.01	<−0.01	−0.9
Summer	High	3.49 **	−4.9 ***	3.9 **	<−0.01	<−0.01
Summer	Spray	0.04	<−0.01	1.5	<−0.01	<−0.01
Autumn	Mid	1.25	−4.1 ***	1.6	1.7	<−0.01
Autumn	High	−0.35	<−0.01	0.9	3.9 **	<−0.01
Autumn	Spray	−4.41 ***	<−0.01	−0.9	2.7 *	<−0.01
Winter	Mid	1.24	−3.8 **	2.4	1.2	<−0.01
Winter	High	−0.33	−4.5 ***	5.0 ***	1.6	<−0.01
Winter	Spray	−4.31 ***	<−0.01	2.7 *	1.9	<−0.01

Asterisks denote statistical significance: * *p* < 0.05; ** *p* < 0.01; *** *p* < 0.001.

## Data Availability

Reported results can be found at ncbi (https://www.ncbi.nlm.nih.gov/, accessed on 20 December 2021), accession numbers OM141383-OM141394 and OM215205-OM216838; see Table S2 for more details.

## Supplementary Materials

The following are available online at https://www.mdpi.com/article/10.3390/plants11050605/s1, Table S1: Abiotic environmental variables at each sampling site. Data were downloaded from Bio-ORACLE (www.bio-oracle.org), Table S2: Bladed Bangiales specimens sampled for this study. Season, locality, intertidal elevation, morphological traits, GenBank accession numbers, and percentage of identity between each sequence and the best BLAST in GenBank are given.
