# Supplementary material for: More than What Meets the Eye: Differential Spatiotemporal Distribution of Cryptic Intertidal Bangiales
Source: Plants (Basel). 2022 Feb 24;11(5):605. doi: 10.3390/plants11050605 (PMC8912569; doi:10.3390/plants11050605)
Supplement: Supplementary file 1 [file plants-11-00605-s001.zip › plants-1520496-supplementary-Table S1.pdf]

Supplementary Table S1. Abiotic environmental variables at each sampling site. Data were downloaded from Bio-ORACLE (www.bio-oracle.org).

Variable	Unit	Statistic	San Carlos	Los Liles	Niebla	Pilolcura	Melinka
Current velocity	m s^{-1}	Max	0.398	0.398	0.353	0.255	0.095
		Mean	0.211	0.211	0.186	0.153	0.058
		Min	0.082	0.082	0.081	0.052	0.015
Salinity	PSU	Max	34.122	34.122	34.131	34.140	33.033
		Mean	33.489	33.489	33.485	33.496	32.631
		Min	32.848	32.848	32.833	32.832	32.198
Temperature	ºC	Max	15.192	15.192	15.080	15.233	13.883
		Mean	12.151	12.151	12.065	12.191	10.750
		Min	9.539	9.539	9.510	9.570	8.616
Photosynthetically active radiation (PAR)	µE m^{-2} d^{-1}	Max	59.362	59.362	64.024	58.106	47.588
		Mean	33.759	33.759	31.385	36.555	27.797
